# Knee instruments and rating scales designed to measure outcomes

**DOI:** 10.1007/s10195-011-0177-4

**Published:** 2012-01-25

**Authors:** E. Carlos Rodriguez-Merchan

**Affiliations:** 1 Department of Orthopaedic Surgery, La Paz University Hospital, Paseo de la Castellana 261, 28046 Madrid, Spain; 2 School of Medicine, Autonomous University, Madrid, Spain

**Keywords:** Knee, Instruments, Rating scales, Outcomes

## Abstract

In this article, the knee instruments and rating scales that are designed to measure outcomes are revised. Although the International Knee Documentation Committee Subjective Knee Form can be used as a general knee measure, no instrument is currently universally applicable across the spectrum of knee disorders and patient groups. Clinicians and researchers looking to use a patient-based score for measurement of outcomes must consider the specific patient population in which it has been evaluated. The Western Ontario and McMaster Universities Osteoarthritis Index is recommended for the evaluation of treatment effect in persons with osteoarthritis (OA). This is a generic health status questionnaire that contains 36 items, is widely used, and easy to complete. The Knee Injury and Osteoarthritis Outcome Score (KOOS) questionnaire evaluates the functional status and quality of life (QoL) of patients with any type of knee injury who are at increased risk of developing OA; i.e., patients with anterior cruciate ligament (ACL) injury, meniscus injury, or chondral injury. So far, the KOOS questionnaire has been validated for several orthopedic procedures such as total knee arthroplasty, ACL reconstruction, and meniscectomy. The utilization of QoL questionnaires is crucial to the adequate assessment of a number of orthopedic procedures of the knee. The questionnaires are generally well accepted by the patients and open up new perspectives in the analysis of prognostic factors for optimal QoL of patients undergoing knee surgery.

## Introduction

There is consensus that patient-reported outcomes have additional value compared to clinical variables when evaluating patient health [1]. The underlying principle is that functional status and quality of life (QoL) can be better described by the patients themselves than by orthopedic surgeons [2].

The Western Ontario and McMaster Universities (WOMAC) osteoarthritis index is recommended for the evaluation of treatment effect in persons with osteoarthritis (OA). It was developed for the elderly with OA, and assesses pain, function, and stiffness in daily living [3–5].

Traumatic knee injuries often cause damage to cartilage, ligaments, and menisci, and may lead to the early development of OA. The Knee Injury and Osteoarthritis Outcome Score (KOOS) covers both the short and long-term consequences of an injury of the knee [6]. The Short Form 36 (SF-36) Health Survey is a generic health status questionnaire that contains 36 items. It measures eight dimensions and is widely used.

The development of QoL instruments has made it possible to obtain an objective assessment of the impact of surgical procedures that takes into consideration physical, psychological, and social aspects of the patient’s everyday activities.

The purpose of this review article is to revise knee instruments and rating scales that are designed to measure outcomes.

## Knee instruments and rating scales

The most widely used disease-specific questionnaire is the WOMAC Osteoarthritis Index. Using visual analog scales, its 24 items probe three dimensions—pain (5 items), stiffness (2 items), and functional difficulty (17 items)—that are judged to be important by such patients. The total score (*n* = 23 items) and the dimension scores (range: 0–100, with 100 indicating the worst possible state) correspond to the sum of the related items divided by the total number of items considered. The WOMAC questionnaire is well recognized for its good validity, reliability, and responsiveness [4, 7–12].

In the past 20 years, there has been considerable growth in the number of knee instruments and rating scales that are designed to measure outcomes from the perspective of the patient. Only a few of these instruments have been evaluated for reliability, validity, and responsiveness. Wang et al. [13] examined the psychometric evidence for patient-reported outcome measures for the knee, and identified the best scores for specific knee conditions. Based on the psychometric data, recommendations included the Cincinnati Knee Rating System, the KOOS, and the Lysholm Knee Score for anterior cruciate ligament (ACL) injuries, the Kujala Anterior Knee Pain Scale for anterior knee pain, the International Knee Documentation Committee (IKDC) Subjective Knee Form, the KOOS, and the Lysholm Knee Score for focal chondral defects, the Western Ontario Meniscal Evaluation Tool (WOMET) for meniscal injuries, and the KOOS for OA. Although the IKDC can be used as a general knee measure, no instrument is currently universally applicable across the spectrum of knee disorders and patient groups. Clinicians and researchers looking to use a patient-based score measure outcomes must consider the specific patient population in which it has been evaluated. Using a diagnostic algorithm that measures the anatomic parts of the knee as separate constructs may solve this dilemma, allowing for the measurement of treatment outcomes across patient groups and the selection of the optimal clinical intervention.

Population data on mortality and life expectancy are generally available for most countries. However, no longitudinal data based on the health-related quality of life (HRQoL) outcome from the HRQoL (EQ-5D) instrument have been reported for orthopedic patients. Jansson and Granath [14] assessed the effect of orthopedic surgery as measured by EQ-5D. In most patients, the EQ-5D (index) score improved but did not reach the level reported for an age- and sex-matched population sample. The results of Jansson and Granath can be used as part of the preoperative patient information to increase the level of patient awareness and cooperation, and to facilitate rehabilitation. In future it will be possible—but not easy—to use the EQ-5D instrument as a complementary consideration in clinical priority assessment.

Rademakers et al. [15] explored which quality aspects (structure, process, outcome) most strongly determine patients’ overall assessment of healthcare, and whether there is variation between different types of patient groups in this respect. Secondary analyses were undertaken on survey data from patients who underwent knee surgery. In these analyses, the patient-given global rating served as the dependent variable, and experiences regarding structure (waiting times, continuity of care), process (doctor–patient communication and information), and outcome (improvement or worsening of symptoms) served as independent variables. Experiences regarding process aspects explained most of the variance in the global rating, followed by structure aspects. Experiences regarding outcome did not explain much variance in the global rating in any of the patient groups. The patient groups did not differ with respect to the type of quality aspects that most predicted the overall assessment. Improving process and structure aspects of healthcare is most likely to increase patients’ overall evaluation of the quality of care as expressed in a global rating. A more sophisticated method of patient-reported outcome measurement, with pre- and post-treatment questionnaires and the inclusion of quality-of-life criteria, might lead to higher associations between outcome and the overall evaluation of the care received.

### Short Form 36 (SF-36)

This is a generic health status questionnaire that contains 36 items. It measures eight dimensions (bodily pain, physical function, social function, role limitations because of physical problems, role limitations because of emotional problems, mental health, vitality, general health perceptions). It is widely used and easy to complete [2, 16].

The SF-36 questionnaire is an HRQoL outcome measure with good metrologic properties [7, 10–12, 17–20]. Health dimension scales are usually computed as described and are combined to obtain summary indices: the Physical Component Summary (PCS), Mental Component Summary (MCS), and Arthritis-Specific Health Index (ASHI) [21–23]. Scores resulting from the summary indices vary from 0 to 100, with higher scores indicating the most favorable state of health.

### KOOS questionnaire

This questionnaire includes the WOMAC Osteoarthritis Index LK 3.0 in its complete and original format, and WOMAC scores can be calculated [3, 4]. The WOMAC is used worldwide for elderly patients with knee OA [3]. The KOOS questionnaire evaluates the functional status and QoL of patients with any type of knee injury who are at increased risk of developing OA; i.e., patients with ACL injury, meniscus injury, or chondral injury. So far, the KOOS questionnaire has been validated for several orthopedic procedures such as total knee arthroplasty (TKA) [24], ACL reconstruction [25], and meniscectomy [26].

### Activities of Daily Living Scale of the Knee Outcome Survey

Irrgang et al. [27] tried to demonstrate the reliability, validity, and responsiveness of the Activities of Daily Living Scale of the Knee Outcome Survey, a patient-reported measure of functional limitations imposed by pathological disorders and impairments of the knee during activities of daily living. The study comprised 397 patients; 213 were male, 156 were female, and the gender was not recorded for the remaining 28. The mean age of the patients was 33.3 years (range, 12–76 years). The patients were referred to physical therapy because of a wide variety of disorders of the knee, including ligamentous and meniscal injuries, patellofemoral pain, and osteoarthritis. The Activities of Daily Living Scale was administered four times during an eight-week period: at the time of the initial evaluation and after 1, 4, and 8 weeks of therapy. Concurrent measures of function included the Lysholm Knee Scale and several global measures of function. The subjects also provided an assessment of the change in function, with responses ranging from greatly worse to greatly better, at 1, 4, and 8 weeks. The Activities of Daily Living Scale was administered to an additional sample of 52 patients (32 male and 20 female patients with a mean age of 31.6 years) before and after treatment within a single day to establish test–retest reliability. Factor analysis revealed two dominant factors: one that reflected a combination of symptoms and functional limitations, and the other only symptoms. The internal consistency of the Activities of Daily Living Scale was substantially higher than that of the Lysholm Knee Scale, resulting in a smaller standard error of measurement for the former scale. Validity was demonstrated by moderately strong correlations with concurrent measures of function, including the Lysholm Knee Scale and the global assessment of function as measured on a scale ranging from 0 to 100 points. Analysis of variance with repeated measures revealed significant improvements in the score on the Activities of Daily Living Scale during the 8 weeks of physical therapy; post hoc testing indicated that the change in the score at 8 weeks was significantly greater than the change at 4 weeks, and that the change at 4 weeks was significantly greater than that at 1 week. As had been hypothesized, the patients in whom the knee had somewhat improved had a significantly smaller change in the score, both at 4 weeks and at 8 weeks, compared with those in whom the knee had greatly improved. The test–retest reliability coefficient was 0.97. These results suggest that the Activities of Daily Living Scale is a reliable, valid, and responsive instrument for the assessment of functional limitations that result from a wide variety of pathological disorders and impairments of the knee.

### Other QoL questionnaires

The Nottingham Health Profile (NHP) and the Arthritis Impact Measurement Scale 2 (AIMS2) are two other important tools that need to be taken into account. Mainard et al. [28] found a clear improvement in QoL, mainly due to physical and psychological dimensions, after TKA using the aforementioned scores.

### Total knee arthroplasty

Kageyama et al. [29] have shown that rheumatoid arthritis (RA) patients with multiple arthroplasties in the lower extremities improve their QoL. However, these patients are still afflicted with secondary diseases derived from RA and experience complications that could shorten their lifespan. Miner et al. [30] found that WOMAC pain and function scores at 12 months after TKA were both correlates of patient satisfaction and perceived improvement in QoL, but knee flexion was not. When assessing these outcomes, WOMAC function appeared to be more important than knee flexion.

Moffet et al. [7] studied the effectiveness of intensive rehabilitation on functional ability and QoL after first TKA. They analyzed the effectiveness of intensive rehabilitation on functional ability and QoL after first TKA. They compared a group who underwent an intensive functional rehabilitation (IFR) program and a control group who received standard care, evaluating the functional ability (WOMAC) and the QoL (SF-36) of all the participants. The main conclusion was that IFR was effective at improving the short-term and mid-term functional ability after uncomplicated primary TKA. More intensive rehabilitation should be promoted in the subacute recovery period after TKA to optimize functional outcomes in the first year after surgery.

In a Dutch study, the KOOS questionnaire showed good internal consistency for all study groups [1]. Reliability was also good in the mild and moderate OA group and the revision TKA group. The KOOS questionnaire seems to be suitable for patients with mild and moderate OA and for patients with a primary TKA. KOOS had a lower construct validity for patients with severe OA on a waiting list for TKA and patients after revision of TKA. However, the construct validity was only assessed by comparing it with the SF-36 and the visual analog scale (VAS) for pain, not with a knee-specific questionnaire. Further validation studies of the KOOS should include knee-specific questionnaires to assess the construct validity.

Inpatient satisfaction with care is a standard indicator of the quality of care delivered during hospitalization. TKA for OA are among the most successful orthopedic interventions that have a positive impact on HRQoL. Baumann et al. [31] evaluated the effect of satisfaction shortly after hospital discharge on 1-month, 6-month and 1-year Medical Outcomes Study 36-item Short Form (SF-36) scores for OA patients after TKA, controlling for patient characteristics, clinical presentation, and preoperative SF-36 scores. The main conclusion was that besides being a quality-of-care indicator, immediate postoperative patient satisfaction with care may lead to new insights into clinical practice, as it is a predictor of self-perceived health status after surgery.

Patient psychological factors have been linked to HRQoL outcomes after total joint replacement (TJR). González Sáenz de Tejada et al. [32] evaluated the relationship between patient expectations before TJR, their fulfillment, and HRQoL outcomes at 3 and 12 months after surgery. Consecutive patients preparing for TJR of the knee or hip due to primary osteoarthritis in 15 hospitals in Spain were recruited for the study. Patients completed questionnaires before surgery and 3 and 12 months afterward: five questions about expectations before surgery and their fulfillment at 3 and 12 months, three HRQoL instruments—the Western Ontario and McMaster Universities Osteoarthritis Index (WOMAC), Short Form 12 (SF-12), and European Quality of Life Instrument (EQ-5D), and questions about sociodemographic information. Student’s *t* test was used to assess the relationship between fulfillment of expectations and gains in HRQoL. Preintervention expectations for TJR ranged from 85 to 86% of patients with high expectations for pain relief and ability to walk to 70% with high expectations about interacting with others. Patients who reported having fulfilled their expectations at 3 and 12 months had significantly greater gains in HRQoL than those who did not. Besides, the authors observed a statistically significant improvement in the percentage of patients who fulfilled their expectations from 3 to 12 months. Patients have high expectations for the benefits of TJR, and those who fulfill their expectations have greater gains in HRQoL as assessed by SF-12, WOMAC, and EQ-5D. Health-care providers should help their patients develop realistic expectations about the impact of TJR.

Gonarthrosis is the most frequent indication to perform arthroplasty of the knee joint. Bugala-Szpak et al. [33] examined the effect of selected factors on QoL evaluation in patients after a knee arthroplasty for gonarthrosis. Forty patients aged 40–85 years (mean age 71.2 years) who underwent knee arthroplasty were examined. KOOS and Short Form-36 (SF-36) questionnaires were used to assess the QoL of the patients. The questionnaires were completed by patients twice: 1–3 days before the operation and 6 weeks post-surgery. Age, gender, BMI, preoperative knee joint range of motion and limb axis, the presence of other implants, and the presence of a knee contracture before surgery were analyzed. The analysis demonstrated that sex, age, presence of other implants, axis, and a preoperative knee contracture did not significantly influence questionnaire scores. As regards the range of knee flexion, outcomes after the arthroplasty were significantly better in patients with preoperative ranges below 90 masculine than in patients with preoperative ranges above 90 masculine. BMI had a significant influence. The main conclusion was that BMI value and range of knee flexion before the arthroplasty significantly influenced the QoL after knee arthroplasty, whereas gender, age, the presence of an additional endoprosthesis, or preoperative joint deformity did not.

In many healthcare systems, people with severe joint disease wait months to years for joint replacement surgery. Empirical data on the health consequences of this delay are scarce, and it is unclear whether people with substantial morbidity upon entry to the waiting list continue to deteriorate further while awaiting surgery. Ackerman et al. [34] investigated changes in HRQoL, health status, and psychological distress among people waiting for TKR surgery. The main conclusion was that, despite substantial initial morbidity, over half of the participants awaiting joint replacement experienced a deterioration in HRQoL during the waiting period. These data provide much-needed evidence to guide health professionals and policymakers in the design of care pathways and resource allocation for people who require joint replacement surgery.

Although the HRQoL for patients who are obese seems to improve after TKA, the magnitude of improvement and the associated factors remain controversial. Nuñez et al. [35] previously found that body mass index was not associated with changes in HRQoL after TKA. Nuñez et al. tried to determine which patient characteristics and surgical factors were associated with worse health status after TKA in patients who are severe or morbidly obese. For patients with knee osteoarthritis who were severe or morbidly obese, various lower limb anthropometric features, degree of IOD, and postoperative complications negatively influenced postoperative WOMAC scores.

### Anterior cruciate ligament

Salavati et al. [36] tried to validate the KOOS for the assessment of competitive athletes with higher-level sports activities after ACL reconstruction. This study illustrated the validity and reliability of the KOOS in measuring the functional status and QoL of athletes after ACL reconstruction. It further validated the use of the KOOS in highly competitive athletes for research on knee injuries.

Recently, the patient’s own evaluation has become an important complement to post-operative clinical assessments. For many patients, there is a change in their life situation after an ACL reconstruction, which may affect the HRQoL in many ways. Mansson et al. [37] evaluated the results in terms of HRQoL 2–7 years after an ACL reconstruction and compared the results with a gender- and age-matched control group. Furthermore, they compared the results for males and females using either the bone-patellar tendon-bone autograft (BPTB) or hamstring tendon autograft (HT). There were no significant differences between males and females. After ACL reconstruction, the patients reported good HRQoL in comparison with a matched sample of the general population. Incorporating non-disease-specific health assessment measures is important in order to further refine disease-specific outcome measurements when evaluating the effect of treatments and attempting to provide cost-effective treatment algorithms.

Borsa et al. [38] tried to determine whether performance-based or patient-reported measures of function are more effective at estimating disability in individuals with an ACL-deficient knee. Subjective rating of knee function was used as the criterion measure for disability, and selected performance-based and patient-reported measures were used as estimation variables. Twenty-nine individuals with an ACL-deficient knee participated in this investigation. Step-wise regression analysis revealed that the Cincinnati Knee Scale, the Lysholm Knee Scale, and the hop index were the most effective estimates of disability. The results demonstrate that patient-reported measures are more closely related to the patient’s level of disability in individuals with an ACL-deficient knee. More research is necessary to substantiate these findings.

Ross [39] assessed the relationship between functional levels in activities of daily living and sports and fear-avoidance beliefs in patients with a history of ACL reconstruction, after controlling for injury-related variables and physical impairment measures. Forty-eight subjects (age 20.6 ± 1.2 years), at a mean of 31.7 ± 16.2 months following ACL reconstruction, participated in this study. Functional levels in activities of daily living and sports were assessed with the Knee Outcome Survey (KOS) Activities of Daily Living Scale (ADLS) and Sports Activity Scale (SAS). Fear-avoidance beliefs were assessed with the physical activity subscale of the Fear-Avoidance Beliefs Questionnaire (FABQ), which was adapted for use in patients with knee pathology. Injury-related variables included whether or not additional knee surgery was performed after the initial ACLR, and the number of months from the most recent ACLR to participation in this study. Physical impairment measures included single-leg hop capabilities, quadriceps strength, and anterior knee joint laxity. Hierarchical linear regression analysis revealed that scores on the physical activity subscale of the FABQ contributed significantly to the KOS ADLS and SAS scores after accounting for injury-related variables and physical impairment measures. The final regression model accounted for 61% of the variance in the KOS ADLS and SAS scores. These results suggest that fear-avoidance beliefs following ACL reconstruction can potentially adversely influence functional levels in activities of daily living and sports.

## Conclusions

The utilization of instruments and rating scales is paramount for the adequate assessment of a number of orthopedic procedures of the knee, including ACL reconstruction, meniscectomy, and TKA. Based on psychometric data, recommendations include the Cincinnati Knee Rating System, the KOOS, and the Lysholm Knee Score for ACL injuries, the Kujala Anterior Knee Pain Scale for anterior knee pain, the IKDC Subjective Knee Form, the KOOS, and the Lysholm Knee Score for focal chondral defects, the WOMET for meniscal injuries, and the KOOS for OA. Although the IKDC Subjective Knee Form can be used as a general knee measure, no instrument is currently universally applicable across the spectrum of knee disorders and patient groups. Clinicians and researchers who are looking to use a patient-based score to measure outcomes must consider the specific patient population in which it has been evaluated.
